# Birt-Hogg-Dubé Syndrome and Hereditary Leiomyomatosis and Renal Cell Carcinoma Syndrome: An Effective Multidisciplinary Approach to Hereditary Renal Cancer Predisposing Syndromes

**DOI:** 10.3389/fonc.2021.738822

**Published:** 2021-09-16

**Authors:** Mohammad Al-Shinnag, Helen Marfan, Rachel Susman, Jan Wakeling, Sonja Gustafson, Simon Wood, Andrew John Mallett

**Affiliations:** ^1^Faculty of Medicine, The University of Queensland, Herston, QLD, Australia; ^2^Genetic Health Queensland, Royal Brisbane and Women’s Hospital, Herston, QLD, Australia; ^3^Department of Medical Imaging, Royal Brisbane and Women’s Hospital, Herston, QLD, Australia; ^4^Department of Medical Imaging, Princess Alexandra Hospital, Woolloongabba, QLD, Australia; ^5^Department of Urology, Princess Alexandra Hospital, Woolloongabba, QLD, Australia; ^6^Department of Renal Medicine, Townsville University Hospital, Douglas, QLD, Australia; ^7^College of Medicine and Dentistry, James Cook University, Douglas, QLD, Australia; ^8^Institute for Molecular Bioscience, The University of Queensland, St Lucia, QLD, Australia

**Keywords:** renal cell cancer, Birt-Hogg-Dubé syndrome, hereditary leiomyoma and renal cell carcinoma syndrome, surveillance, multidisciplinary (care or team)

## Abstract

**Aim:**

We aimed to describe and analyse clinical features, characteristics, and adherence to surveillance guidelines in an Australian Birt-Hogg-Dubé syndrome (BHD) and hereditary leiomyomatosis and renal cell cancer (HLRCC) cohort.

**Methods:**

All identified patients with a diagnosis of BHD or HLRCC at RBWH 01/01/2014-01/09/2019 were included (HREC/17/QRBW/276). All patients were initially assessed and counselled by a clinical geneticist and then referred to an adult nephrologist. Baseline and incidental clinical variables were extracted and analysed.

**Results:**

Fifty-seven patients were identified (28 BHD, 29 HLRCC) with a median age of 47 years. The median and cumulative follow-up were 1 and 99 years, respectively. Baseline renal MRI occurred in 40/57 patients, and 33/57 had regular MRI as per the national guidelines (eviQ). Of 18/57 without baseline imaging, nine were yet to have imaging, seven were lost follow-up, and two patients had logistic difficulties. RCC was diagnosed in 11/57 patients: two of 28 with BHD were diagnosed with RCC aged 73 and 77, both prior to commencement of surveillance. Nine of 29 patients with HLRCC were diagnosed with RCC (one of 29 during surveillance at 47 years of age) and eight of 29 prior to commencement of surveillance (11–55 years). Amongst BHD patients, cutaneous fibrofolliculomas were noted in 15 patients, lung cysts were detected in seven patients, spontaneous pneumothoraces in five patients, and parotid oncocytoma in two of 28. Amongst those with HLRCC, cutaneous leiomyomas were noted in 19/29, cutaneous leiomyosarcoma diagnosed in one of 29, and uterine fibroids in 13 female patients.

**Conclusion:**

Evidence-based RCC screening in BHD and HLRCC cohort is feasible and able to identify incidental renal lesions. Multidisciplinary patient management enables expedited genetic counselling, diagnosis, longitudinal screening, and RCC management. The success of this clinical model warrants consideration of undertaking longitudinal screening of BHD and HLRCC patients by nephrologists.

## Background

It is estimated that hereditary forms of renal cell cancer (RCC) account for 2%–4% of renal cancer (1). Within that small proportion of all cases of RCC, there are many monogenic disorders that have been linked to RCC, including BHD and HLRCC (**Table 1**).

**Table 1:** Diagnostic criteria for Birt-Hogg-Dube syndrome and hereditary leiomyomatosis and renal cell cancer syndrome.

Birt-Hogg-Dube syndrome	
Major criteria	At least 5 adult onset fibrofolliculomas or trichodiscoma, at least 1 confirmed on histology
	Pathogenic *FLCN* germline pathogenic variant
Minor criteria	Multiple pulmonary cysts, with or without spontaneous primary pneumothorax
	Renal cancer (early onset <50 years) or multifocal or bilateral renal cancer or renal cancer of mixed chromophobe and oncocytic histopathology
	A first-degree relative with BHD
Hereditary leiomyomatosis and renal cell cancer syndrome	
Major criteria	Multiple cutaneous piloleiomyomas, histopathologically confirmed
Minor criteria	Surgical treatment for severely symptomatic uterine leiomyomas before age 40
	Type 2 papillary renal cell carcinoma before age 40
	A first‐degree family member who meets 1 of the abovementioned criteria

BHD is caused by heterozygous germline pathogenic variants in *FLCN*-encoding folliculin. It is inherited in an autosomal dominant pattern, characterised by skin fibrofolliculomas, multiple lung cysts, spontaneous pneumothorax, and RCC (2). Previously, over 600 families with pathogenic *FLCN* variants have been reported worldwide (3), indicating its significant rarity and also the need for further research and characterisation. The diagnosis of BHD can be suspected in the presence of one major or two minor criteria as proposed by Menko et al. (2), but a definitive diagnosis requires the identification of a pathogenic *FLCN* variant (4 **).**

The most threatening potential complication of BHD is the incidence of RCC. It is estimated that up to 19% of patients with BHD develop RCC at an average age of 50 years (5, 6) (3). Patients with *FLCN* pathogenic variants have sevenfold higher risk of developing RCC than the general population (7). The true lifetime risk of developing RCC in patients with BHD is uncertain due to several reasons. Firstly, diagnosis of BHD can be based on dermatological manifestations and the risk of developing RCC may not be similar amongst all affected families (2, 8). Secondly, RCC in BHD is often bilateral or multifocal; however, in a substantial number of cases, it is not recognised on initial diagnosis and not until the detection of concurrent RCC lesions (9).

The recommendations of the Australian National Guidelines (eviQ) regarding cancer risk management for BHD is to obtain a baseline abdominal MRI at the age of 20 years and if no abnormality is detected to then arrange further MRI imaging every 3 years or high-resolution ultrasound imaging for life (10). Although abdominal MRI is the preferred imaging modality, high-resolution ultrasound is a reasonable alternative if MRI is not available.

Hereditary leiomyomatosis and renal cell cancer (HLRCC or Reed’s syndrome) is similarly an autosomal dominant disorder caused by heterozygous germline pathogenic variants in *FH* which encodes for the enzyme fumerate hydratase (FH). This enzyme (FH) has a major role in the tricarboxylic acid cycle and acts as a tumour suppressor (11). It has a very low or undetectable activity level in tumours from individuals with leiomyomatosis (12). HRLCC has three main characteristic clinical features: skin piloleiomyomas, uterine leiomyomas (fibroids) at early age, and RCC (11). Clinical diagnostic criteria for HLRCC were proposed by Smit et al., and the diagnosis is more likely when a proband meets the major criteria and it is suspected when a proband meets at least two minor criteria (13).

FH-deficient RCC (also known as HLRCC-associated RCC) is a well-recognised renal cell tumour type (14, 15) which has a distinct histological appearance including characteristic large nuclei with a very prominent organophilic or eosinophilic nucleolus surrounded by a clear halo in addition to FH deficiency on immunohistochemistry. These tumours are predominantly unilateral, solitary, and aggressive with early preponderance to metastasis (16). The estimated lifetime risk for RCC in HLRCC is 15% with mean age at diagnosis being 41 years and 7% of RCC has been diagnosed when younger than 20 years (11). There are varying recommendations about when to start the surveillance for patients with HLRCC and how often screening should be conducted. There is practical consensus that MRI with 3 mm slices throughout the kidneys on annual basis is the optimal imaging modality.

Whilst these broad characterisations are clinically useful, description of individual experience and clinical translation into longitudinal healthcare is lacking. There is a need to better identify and characterise meaningful cohorts of such patients with rare disease in order illuminate understandings and knowledge. In this setting, we sought to identify known cases of BHD and HLRCC within our health service in order to better describe their characteristics and outcomes.

## Methods

Medical records at Royal Brisbane and Women’s Hospital were screened for patients with a clinical or genetic diagnosis of BHD or HLRCC through the period 1st January 2014 to 1st September 2019. Ethics approval was sought and granted (HREC/17/QRBW/276). For the identification of BHD cases, the search inclusion criteria were for patients with pathogenic/likely pathogenic germline variants in *FLCN* gene, a clinical diagnosis of BHD or individuals at 50% risk of BHD. The inclusion criteria for HLRCC were confirmed pathogenic/likely pathogenic variant in *FH* gene or 50% risk of being a carrier. Patients without clinical and or histopathological findings to suggest BHD or HLRCC, patients without identified variants (variant of uncertain significance, pathogenic or likely pathogenic) in *FLCN* or *FH*, or family history of the disease (BHD or HLRCC) were excluded.

Medical records of identified patients fulfilling the inclusion criteria were reviewed (including clinical letters, clinic notes, and genetic testing reports) with data extracted. Furthermore, all identified patients had been previously referred to the state clinical genetic service, Genetic Health Queensland (GHQ) and had been assessed by a clinical geneticist before then being referred to an adult nephrologist for ongoing clinical assessment and surveillance.

Baseline clinical variables were extracted from patient medical records (including: date of birth, age at diagnosis, gender, how was the diagnosis made, genetic testing results, clinical features of the disease, family history, diagnosis of RCC (age at onset and how was diagnosed), surveillance imaging (modality and frequency), incidental findings, and false-positive findings on imaging. One of the main aims of this study was to assess the adherence to the recommended national surveillance guidelines following the diagnosis and referral to the nephrology unit for ongoing follow-up.

## Results

A total of 28 patients with BHD and 29 patients with HLRCC met the inclusion criteria and were included in the study. Their characteristics and outcomes (**Table 2**) are stratified by diagnosis as follows:

**Table 2:** Cohort characteristics.

a. HLRCC subcohort.								
**Identifier**	Proband *vs*. predictive	*FH* gene variant	Classification	Gender	Clinical features	Age at dx	RCC (age)	Histology
H1	Proband	*FH*: c.178_180delinsTT, p.Lys61fs	P	Female	Cutaneous leiomyomas and uterine fibroids, family history of HLRCC (sibling with RCC)	46	Yes (47)	HLRCC-associated RCC (papillary, tubular, and cribriform features)
H2	Predictive	*FH*: c.178_180delinsTT, p.Lys61fs	P	Female	Asymptomatic, family history (mother with RCC and maternal aunt HLRCC)	21	No	
H3	Predictive	*FH*: c.178_180delinsTT, p.Lys61fs	P	Female	Asymptomatic, family history (mother and maternal aunt with HLRCC)	28	No	
H4	Predictive	*FH*: c.320A>C, p. Asn 107Thr	P	Female	Personal history of cutaneous leiomyomatosis and uterine fibroids, and family history (son: FH pathogenic variant)	66	No	
H5	Predictive	*FH*:c.320A>C, p.Asn107Thr	P	Male	Cutaneous leiomyoma and family history of FH pathogenic variant	57	No	
H6	Proband	*FH*: c.320A>C, p. Asn 107Thr	P	Male	Cutaneous Leiomyoma and Family History (mother: cutaneous leiomyomatosis and uterine fibroids)	39	No	
H7	Proband/confirmatory	*FH*:c.413-414del(p.Lue138fs), MEtc.2962C>T	P	Female	Personal History of RCC at 44 years, cutaneous leiomyoma and Family history HLRCC	50	Yes (43)	Multifocal RCC, hybrid features of papillary RCC and clear cell RCC
H8	Proband/confirmatory	*FH*: c.413_414del (p.Leu138fs), MEtc.2962C>T	P	Female	Personal RCC at 19 years and family history HLRCC	25	Yes (19)	Papillary renal cancer type 1
H9	Proband	*FH*: c.413_414del (p.Leu138fs)	P	Female	Personal RCC at 11 years, cutaneous leiomyosarcomas and leiomyomas, uterine leiomyomas and family history	28	Yes (11)	RCC histology not available
H10	Proband	*FH*:668_669delAA, p.Lys223Argfs*26	P	Female	HLRCC-related RCC at the age of 19 years, cutaneous leiomyoma	19	Yes (19)	FH-deficient RCC (HLRCC-related RCC)
H11	Proband	*FH*: c.689A>G, p.Lys230 Arg	P	Female	Clear cell renal cell carcinoma at 36 years, cutaneous leiomyoma, and uterine fibroids	36	Yes (36)	Clear cell RCC
H12	Predictive	*FH*: c.689A>G, p.Lys230 Arg	P	Female	Asymptomatic, family history	33	No	
H13	predictive	*FH*: c.689A>G, p.Lys230 Arg	P	Female	Personal history of uterine fibroid and family history	62	No	
H14	confirmatory	*FH*: c.1027C>T, p. Arg343*	P	Female	Cutaneous leiomyomatosis and uterine fibroids and family history leiomyomas and uterine fibroids	50	No	
H15	Predictive	*FH*: c.1027C>T, p. Arg343*	P	Male	Cutaneous leiomyoma and family history (siblings with cutaneous leiomyomas)	65	No	
H16	Predictive	*FH*: c.1027C>T, p. Arg343*	P	Female	Uterine fibroids and family history	23	No	
H17	Proband	*FH*: c.1180G>C, p.Val394Leu	VUS=>LP	Female	Cutaneous leiomyoma and uterine fibroids and family history	35	No	
H18	Predictive	*FH*: c.1180G>C, p.Val394Leu	VUS=>LP	Female	Cutaneous leiomyoma and uterine fibroid and family history	63	No	
H19	Predictive	*FH:* c. 1277_1287, p.Ala426fs	P	Female	Cutaneous piloleiomyoma and family history of HRLCC (mother: cutaneous leiomyosarcoma)	20	No	
H20	Proband	*FH*:c.1376T>C, p.Leu459Pro	P	Female	Cutaneous leiomyoma, uterine leiomyoma, and family history of cutaneous leiomyoma and fibroids	53	No	
H21	Proband	*FH*: c.1424C>A, p.Ala475Glu	VUS=>LP	Male	FH-deficient RCC at the age of 55 years and cutaneous leiomyoma	62	Yes (55)	FH-deficient HLRCC-related RCC
H22	Proband	*FH*: c.1424C>A, p.Ala475Glu	LP	Female	FH-deficient uterine leiomyoma, cutaneous leiomyoma, and family history	27	Yes (27)	FH-deficient HLRCC-related RCC
H23	Proband	*FH*:c.1445T>G, p.Leu482*	P	Male	Papillary type 2 RCC at 42 years	42	Yes (42)	Papillary type 2 RCC
H24	Predictive	*FH*: c.1445T>G, p.Leu482*	P	Female	Asymptomatic, family history	15	No	
H25	Predictive	*FH*: c.1445T>G, p.Leu482*	LP	Female	Asymptomatic, family history	34	No	
H26	Predictive	*FH*: c1445T>G, p.Lue482*	LP	Male	Asymptomatic, family history	57	No	Adrenal adenoma
H27	Predictive	*FH*: c.1445T>G, p.Leu482*	LP	Female	Uterine fibroids and family history	29	No	
H28	Predictive	*FH*: c.1475_1476delTC, p.Leu492HisfsX6)	P	Male	Probable cutaneous leiomyoma and family history	50	No	
H29	Predictive	*FH*: c.1475_1476delTC, p.Leu492Hisfs*6)	P	Male	Asymptomatic, family history	26	No	
b. BHD subcohort													
Identifier	Proband *vs*. predictive	*FLCN* gene variant	Classification	Gender	Diagnostic features	Age at Dx	Clinical features	RCC (age)	Fibrofolliculoma	Lung cyst	Pneumothrax	RCC	Relevant findings
B1	Proband	*FLCN*: c.249+1_2delGCinsA	P	Female	Fibrofolliculoma (diagnosed by dermatologist), clinical diagnosis	62	Fibrofolliculomas	No	Yes	No	No	No	Hepatic and renal cysts adrenal adenoma
B2	Predictive	*FLCN*: c.249+1_2delGCinsA	P	Male	Family history, predictive testing	35		No	NA	NA	No	No	
B3	Predictive	*FLCN*: c.249+1_2delGCinsA	P	Male	Family history, predictive testing	39	Possible fibrofolliculoma	No	Yes	No	No	No	
B4	Predictive	*FLCN*: c.249+1_2delGCinsA	P	Male	Family history, predictive testing	34	Nil	No	No	No	No	No	Thyroid cystic nodule
B5	Proband	*FLCN*: c.384C>G, p.Ser128Arg	VUS	Female	Renal biopsy: renal oncocytosis *vs*. chromophobe RCC, genetic testing VUS	67	Nil	No	No	No	No	No	Kidney biopsy: renal oncocytosis *vs*. chromophobe RCC
B6	Predictive	*FLCN*: c.469_471delTTC, p.Phe157del	LP	Female	Family history, predictive testing	37	Fibrofolliculomas	No	Yes	No	No	No	
B7	Predictive	*FLCN*: c.469_471delTTC, p.Phe157del	LP	Female	Family history, predictive testing	60	Parotid oncocytoma	No	No	No	No	No	Parotid oncocytoma at age 54
B8	Predictive	*FLCN*: c.469_471delTTC, p.Phe157del	LP	Female	Spontaneous pneumothorax (32 years), family history, predictive testing	34	Pneumothorax	No	No	Yes	Yes	No	
B9	Predictive	*FLCN*: c.469_471delTTC, p.Phe157del	LP	Male	Family history, predictive testing	29	Fibrofolliculomas	No	Yes	No	No	No	
B10	Predictive	No genetic test (obligate carrier)	NA	Male	Obligate carrier (daughter and son confirmed *FLCN* LP variant)	67	Nil	No	No	No	No	No	
B11	Proband	*FLCN:* c.763C>T, pHis255Tyr	LP	Male	Fibrofolliculoma (biopsy proven) and family history, confirmatory testing	38	Fibrofolliculomas	No	Yes	No	No	No	
B12	Proband	*FLCN*: c.1157C>G, p.Ser386*	P	Female	Fibrofolliculoma/trichodiscoma, diagnostic testing found FLCN P variant	77	Fibrofolliculoma, lung cysts	No	Yes	Yes	No	No	
B13	Predictive	*FLCN*: c.1177-5_1177-3-del	P	Male	Family history, predictive testing	46	Nil	No	No	No	No	No	
B14	Proband	*FLCN:* c.1285dupC, p.His429Profs*27	P	Male	RCC, diagnostic testing	81	Fibrofolliculomas	Yes (clear cell RCC) grade 2 at 77 years	Yes	Probable	No	Yes	Parotid oncocytoma (74), bladder CA, personal and FH colonic polyps
B15	Proband	*FLCN*: c.1285dupC, p.His429Profs*27	P	Male	Spontaneous pneumothorax (35 years), family history, and predictive testing	49	Pneumothorax	No	Yes	Yes	Yes	No	
B16	Predictive	*FLCN*: c.1285dupC, p.His429Profs*27	P	Female	Lung cyst, family history (sons and maternal relatives), predictive testing	78	Lung cyst, possible fibrofolliculoma	No	Probable	Yes	No	No	
B17	Predictive	*FLCN*: c.1285dupC, p.His429Profs*27	P	Female	Spontaneous pneumothorax, lung cysts, and family history, confirmatory testing	68	Lung cyst and pneumothorax.	No	Probable	Yes	Yes	No	
B18	Proband	*FLCN*: c.1285dupC, p.His429Profs*27	P	Male	Recurrent pneumothorax (22 and 23 years), lung cysts, fibrofolliculoma, and family history	72	Fibrofolliculoma, lung cysts, and pneumothorax	No	Yes	Yes	Yes	No	Solid renal lesion (left 10 mm), complex renal cyst, multiple simple cysts
B19	Predictive	*FLCN*: c.1285dupC, p.His429Profs*27	P	Female	Family history, predictive testing	61	Fibrofolliculoma	No	Yes	No	No	No	
B20	Predictive	*FLCN*: c.1285dupC, p.His429Profs*27	P	Female	Family history, predictive testing	64	Fibrofolliculoma	No	Probable	Probable	No	No	Emphysema, hemochromatosis, oophorectomy for ovarian torsion
B21	Proband	*FLCN*: c.1318_1334dup, p.Leu449Glnfs*25	P	Female	Fibrofolliculoma (biopsy proven), diagnostic testing: *FLCN* P variant, family history	50	Fibrofolliculoma	No	Yes	No	No	No	
B22	Predictive	*FLCN*: c.1318_1334dup, p.Leu449Glnfs*25	P	Female	Family history, predictive testing	56	Fibrofolliculoma	No	Yes	No	No	No	
B23	Predictive	*FLCN*: c.1318_1334dup, p.Leu449Glnfs*25	P	Female	Family history, predictive testing	39	Fibrofolliculoma	No	Yes	No	No	No	
B24	Predictive	*FLCN*: c.1318_1334dup, p.Leu449Glnfs*25	P	Female	Multiple pneumothoraces, family history, predictive testing	74	Multiple pneumothoraces (40s) renal cysts and fibrofolliculoma	No	No	Yes	Yes	No	
B25	Proband	*FLCN*: c.1333G>A, p.Ala445Thr	VUS	Male	Left nephrectomy: chromophobe carcinoma and RCC (FLC variant unlikely pathogenic)	73		Yes at 73	No	No	No	Yes	
B26	Predictive	No genetic test		Female	Likely fibrofolliculoma and family history (brother had likely pathogenic variant in FLCN (c.763C>T)	38	Possible fibrofolliculoma	No	Yes	No	No	No	Clinical diagnosis based on fibrofollliculoma as per dermatologist and family history
B27	Proband	*FLCN* no P/LP variants detected		Male	Trichodiscomas (histologically confirmed), diagnostic testing: no P/LP variants	54	Trichodiscomas	No	Yes	No	No	No	Hepatic and renal cysts, cellular atypia, secondary to JC viriuria
B28	Predictive	Testing in proband negative		Female	Fibrofolliculomas (histologically confirmed), strong FHx (maternal uncle with chromophobe RCC, maternal cousins ×2 with chromophobe RCC in 30s), son pneumothorax	47	Fibrofolliculomas	No	Yes	No	No	No	FHx chromophobe RCC ×2 cousins

*This is standard HGVS amino acid nomenclature for a nonsense substitution variant.

### BHD

A total of 28 patients with BHD were identified as having received clinical care over the 3-year period from 01/01/2014 to 01/09/2019. Sixteen of 28 were female, and the median age was 56 years. Median clinical follow-up was 1 year with cumulative follow-up being 55 years. Ten of 28 were index proband cases, and 14/28 were clinically and/or genetically affected relatives.

#### Cutaneous Features

In regard to cutaneous manifestations, 16/28 had documented skin-coloured, dome-shaped, papules on one or more of the face, neck, and upper trunk consistent with fibrofolliculoma, 1/28 had trichodiscoma, and 5/28 had skin lesions suspicious for fibrofolliculoma; however, this dermatological diagnosis was not confirmed.

#### Respiratory Features

Baseline chest imaging in the form of chest X-ray or computed tomography (CT) was reviewed for 18/28 (15/18 had chest X-ray and 4/18 had chest CT scan). Within the limits of this imaging, pulmonary cysts were identified in seven individuals. One patient had emphysematous changes on chest CT in the setting of a remote limited smoking history (one pack year in late adolescence), raising the possibility of pulmonary cysts. Five of seven patients with pulmonary cysts had experienced spontaneous pneumothoraces beginning at ages 22, 29, 32, 35, and 40 years. One of five such patients required pleurodesis. In total, five of 28 patients had symptomatic pulmonary involvement; however, the burden of asymptomatic disease was unclear as only four of 28 had undergone screening chest CT imaging which is insufficient to be able to infer such asymptomatic pulmonary involvement. All patients received counselling in regard to smoking abstinence, and no patients entering into longitudinal follow-up were known to be active smokers. Detailed past smoking history and baseline pulmonary function tests were not available.

#### Other Features

Unilateral parotid oncocytoma was reported in two patients at 54 and 57 years, respectively.

#### RCC Diagnoses

RCC was diagnosed in two patients (B14 and B25) prior to the commencement of surveillance, and those diagnoses had resulted in prompt surgical intervention. A male patient (B14) was diagnosed with RCC at the age of 77 years having previously had parotid oncocytoma diagnosed at 74 years, with a confirmed BHD diagnosis on genetic testing occurring at 81 years. Another male patient (B25) who was diagnosed with RCC at 73 years had *FLCN* sequencing, which identified a heterozygous variant of uncertain significance (VUS; c.1333G>A). This patient’s nephrectomy histology demonstrated mixed chromophobe RCC. No incident cases of RCC were identified during screening.

#### Genetic Features

Heterozygous pathogenic or likely pathogenic variants in *FLCN* were confirmed in 23/28 patients (**Table 3**). There were specific circumstances documented for the five of 28 remaining patients (B25 described above, B5, B10, B26, B27) including two of five in whom a *FLCN* VUS was identified (B25, B5).

**Table 3:** Heterozygous *FLCN* variants identified on clinically accredited diagnostic testing.

Gene and transcript	Variant	ACMG classification	Exon/description
*FLCN* NM_144997.5	c.249+1_2delGCinsA	Pathogenic	Exon 5 (intronic) 125 bp deletion, canonical splice site
*FLCN* NM_144997.5	c.384C>G, p.Ser128Arg	VUS	Exon 5 missense
*FLCN* NM_144997.5	c.469_471delTTC, p. Phe157del	Likely pathogenic	Exon 6, in-frame deletion
*FLCN* NM_144997.5	c.763C>T, pHis255Tyr	Likely pathogenic	Exon 7, missense
*FLCN* NM_144997.5	c.1157C>G, p. Ser386*	Pathogenic	Exon 11, nonsense
*FLCN* NM_144997.5	c.1177-5_1177-3-del	Pathogenic	Exon 11, intronic deletion of 3 bp close to conserved splicing acceptor site of exon 11, frameshift cause premature stope codon
*FLCN* NM_144997.5	c.1285dupC, p. His249Profs*27	Pathogenic	Exon 11, 1 bp duplication, frameshift (nonsense)
*FLCN* NM_144997.5	c.1318_1334dup, p.Leu449Glnfs*25	Pathogenic	Exon12, 17 bp duplication, frameshift, nonsense
*FLCN* NM_144997.5	c.1333G>A, p.Ala445Thr	VUS	Exon 12, missense

*This is standard HGVS amino acid nomenclature for a nonsense substitution variant.

A 67-year-old female (B5**)** had a *FLCN* VUS identified (c.384C>G, p.Ser128Arg) but had histological features suggestive of BHD in the form of renal oncocytosis on kidney biopsy as an incidental finding during investigation for an unrelated and unexplained acute kidney injury that resolved. B10 was an obligate carrier who did not undergo confirmatory genetic testing as he was 67 years and had affected maternal relatives, as well as a son and a daughter who had the same familial pathogenic *FLCN* variant as the maternal relatives. B26 had a personal and family history of skin lesions (father and brother) as assessed by a dermatologist who reported features consistent with fibrofolliculoma, however, confirmatory skin biopsy was declined as was *FLCN* germline testing. B27 had multiple cutaneous trichodishomas which was confirmed on histopathology and had *FLCN* gene testing which did not identify any significant variants. B28 had a personal history of fibrofolliculoma and a strong family history in the form of a maternal uncle and two maternal cousins all with chromophobe RCC, and a son with pneumothorax. Genetic testing in an affected relative did not identify any clinically significant *FLCN* variants in B28.

#### Surveillance Adherence

Following an initial assessment by a clinical geneticist who established the diagnosis of BHD, patients were then referred to an adult nephrologist for longitudinal surveillance. Australian guidelines (eviQ) suggest baseline abdominal magnetic resonance imaging (MRI) at 20 years and if no abnormalities detected then three yearly MRI or two yearly high-resolution ultrasound scan. Amongst the 28 patients who were diagnosed with BHD and referred to nephrology, 22 individuals had regular yearly review, two were referred but yet to be reviewed, and four did not attend their nephrology appointments. Eighteen of 28 completed baseline abdominal MRI, one of 28 had CT abdomen as they could not undergo MRI, four of 28 were yet to have their first MRI, and five of 28 did not have a baseline MRI scan. Subsequently, 11/28 had documented regular MRI abdomen at least once every 3 years.

### HLRCC

Twenty-nine patients with HLRCC were identified as having received clinical care over the 3-year period from 01/01/2014 to 01/09/2019. Twenty-one of 29 were female, and the median age was 36 years. Median clinical follow-up was 1 year, and cumulative follow-up was 45 years. Ten of 29 were index proband cases, and 19/29 were diagnosed on predictive testing.

#### Non-RCC Features

Cutaneous leiomyomas were noted in 19/29 patients, cutaneous leiomyosarcoma was diagnosed in one of 29, and uterine leiomyomas in 13/21 female patients.

#### RCC Diagnoses

There were seven patients diagnosed with RCC prior the commencement of surveillance at ages of 11, 19, 19, 27, 36, 43, and 55 years. Three of seven were women from the same family, with two of three of these family members having uterine leiomyomas and cutaneous leiomyomas, and one of three (H9) experiencing cutaneous leiomyosarcoma.

Three of 29 patients had kidney or related lesions identified by screening; two of whom were diagnosed as being RCC. A 47-year-old female patient (H1) had a complex cystic lesion identified on surveillance MRI measuring 26 mm in the lower pole of the left kidney. This was associated with the presence of a prominent left paraaortic lymph node and was suspicious for RCC. Screening MRI 1-year prior had identified simple cortical cysts in the upper and lower poles of the left kidney as well as a simple cyst in the mid-pole of the left kidney. Prompt urology referral resulted in urgent left nephrectomy and extended retroperitoneal lymph node dissection. This confirmed the presence of RCC with metastasis to a single paraaortic lymph node. Although the appearance on the initial MRI was suggestive of simple cysts, this case demonstrates the importance of having a higher index of suspicion for RCC in regard to kidney cystic changes in HLRCC, and the role of an experienced radiologist in assessing kidney radiological findings in HLRCC patients.

A further patient (H23) had a solid lesion on the upper pole of the left kidney detected on CT scan at 42 years and underwent laparoscopic left total nephrectomy prior to commencing annual screening. The histology revealed FH-deficient RCC consistent with HLRCC-associated RCC; however, this finding was upon reappraisal and further investigation as the initial histopathology was thought to be papillary type II RCC. This highlights the importance of involving pathologists with a special interest in HLRCC-related RCC. Recurrence was identified on subsequent annual screening in the form of retrocrural and para-oesophageal lymphadenopathy that was confirmed on FDG-PET scan.

Lastly, a 66-year-old male was found to have a suspicious right kidney 9 mm minimally enhancing lesion which was thought to be solid on imaging. After urology referral, he underwent right partial nephrectomy; however, kidney lesion histopathology revealed a benign cortical cystic lesion without evidence of RCC. He subsequently resumed regular annual MRI surveillance.

#### Genetic Features

Diagnosis of HLRCC was confirmed on initial genetic testing in 25/29 in whom a heterozygous pathogenic (22/29) or likely pathogenic (3/29) *FH* gene variant was detected (**Table 4**). This included one patient (H17) who had a clinical diagnosis of HLRCC in whom a variant was identified (*FH*: c.1180G>C, p. Val394Leu), was initially classified as VUS. She had a sister who also had a clinical diagnosis of HLRCC and her mother (H18) had a probable clinical diagnosis of HLRCC. As the variant cosegregated with disease in affected family members and the variant was previously reported in affected family with HLRCC, therefore this variant was upgraded to likely pathogenic.

**Table 4:** Heterozygous *FH* variants identified on clinically accredited diagnostic testing.

Gene and transcript	Variant	ACMG classification	Exon/description
*FH* NM_000143.3	c.178_180delinsTT, p. Lys61fs	Pathogenic	Nonsense exon 2
*FH* NM_000143.3	c.320A>C, p. Asn 107Thr	Pathogenic	Missense exon 3
*FH* NM_000143.3	c.413-414del (p. Lue138fs)	Pathogenic	Frameshift deletion exone 4
*FH* NM_000143.3	c.668_669delAA, p. Lys223Argfs*26	Pathogenic	Frameshift deletion exon 5
*FH* NM_000143.3	c.689A>G, p. Lys230 Arg	Pathogenic	Missense exon 5
*FH* NM_000143.3	c.1027C>T, p. Arg343*	Pathogenic	Nonsense exon 7
*FH* NM_000143.3	c.1180G>C, p. Val394Leu	VUS=>LP (coseg)	Missense exon8
*FH* NM_000143.3	c. 1277_1287, p. Ala426fs	Pathogenic	Frameshift, exon 9
*FH* NM_000143.3	c.1376T>C, p. Leu459Pro	Pathogenic	Missense, exon 10
*FH* NM_000143.3	c.1424C>A, p. Ala475Glu	VUS=>LP (coseg)	Missense exon 10
*FH* NM_000143.3	c.1445T>G, p. Leu482*	Pathogenic	Nonsense exon 10
*FH* NM_000143.3	c.1475_1476delTC, p. Leu492HisfsX6	Pathogenic	Frameshift deletion exon 10

*This is standard HGVS amino acid nomenclature for a nonsense substitution variant.

Another variant (*FH*: c.1424C>A, p.Ala475Glu) was found in a patient (H22) who was diagnosed with FH-deficient uterine leiomyoma at 26 years. Her father (H21) was diagnosed with FH-deficient RCC at 55 years, and this variant was initially classified as a VUS. Genetic testing for H21 was repeated in another genetic laboratory with special interest in the *FH* gene, which resulted in the VUS being reclassified as likely pathogenic.

#### Surveillance Adherence

Patients who had established diagnosis of HLRCC were referred to an adult nephrologist for baseline abdominal MRI and for surveillance. Twenty-four of 29 referred individuals were reviewed by an adult nephrologist, two of 29 lost to follow-up, two had logistic difficulties due to living in rural regions, and one yet to attend his first review in the renal outpatient clinic. Twenty-two of 29 individuals had their baseline MRI abdomen, 20/29 had regular annual MRI and clinical review, two of 29 did not have their baseline MRI, and five yet to have their MRI.

## Discussion

**Figure 1:**
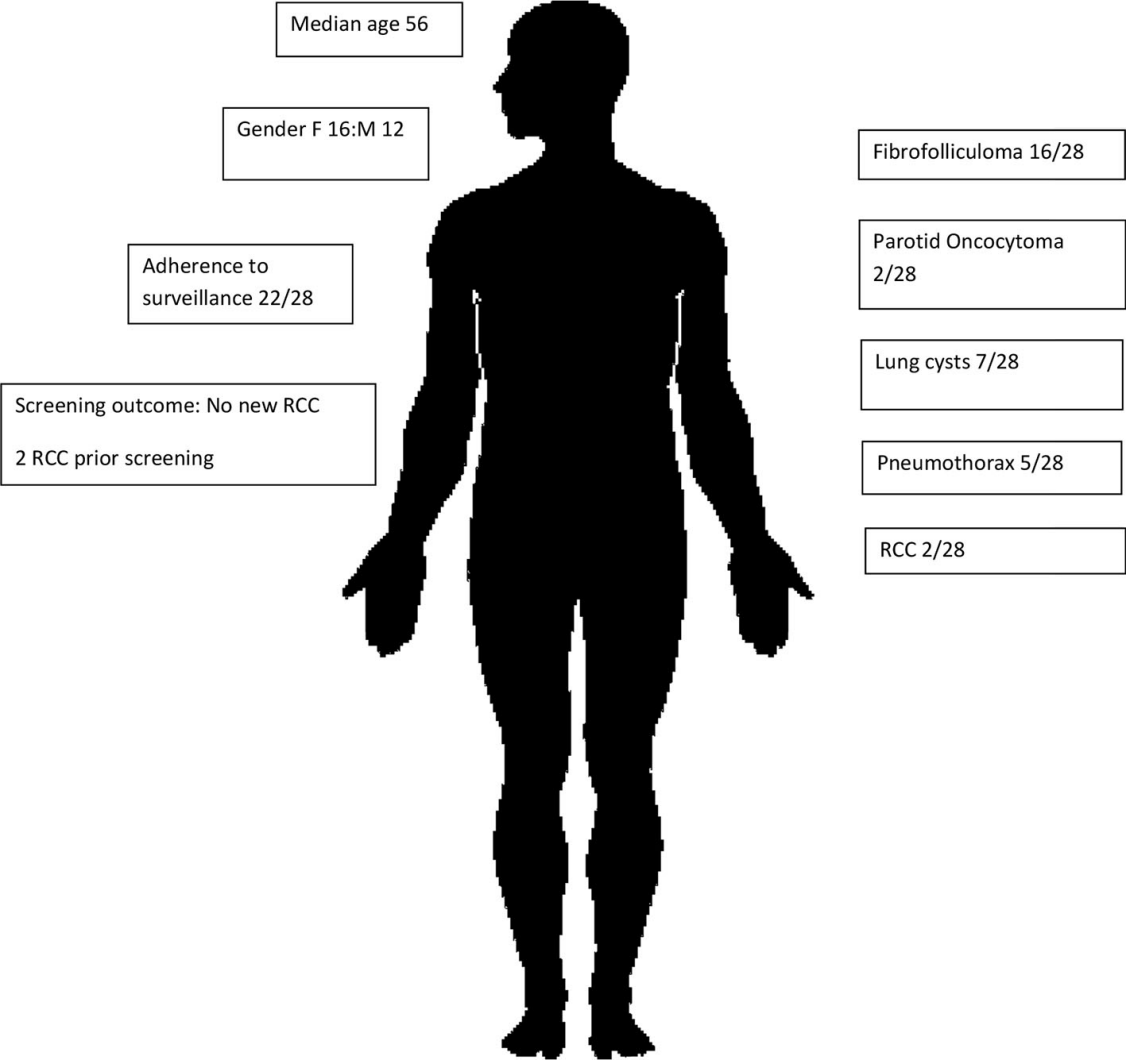
BHD subcohort characteristics.

**Figure 2:**
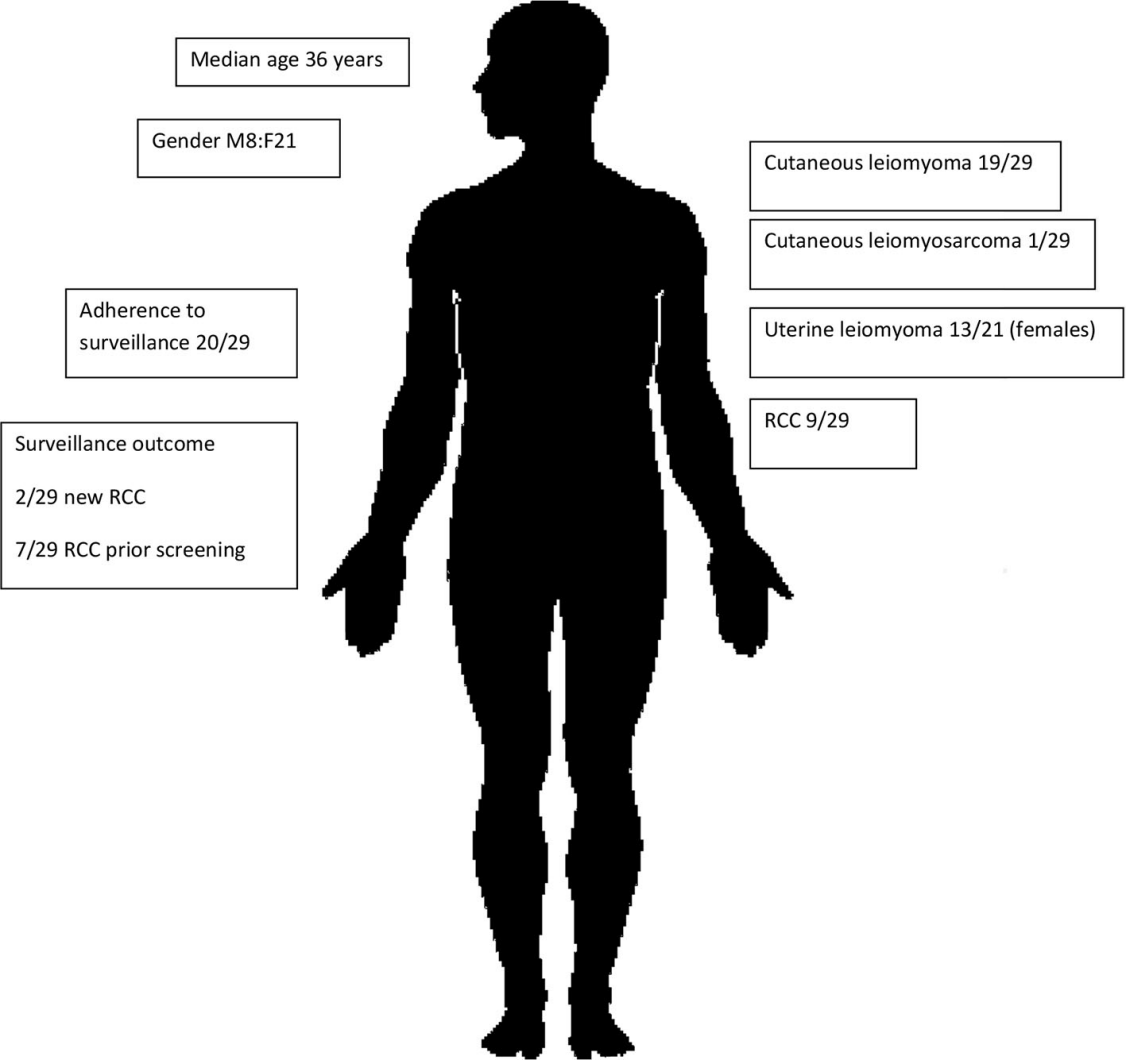
HLRCC subcohort characteristics.

This is the first and largest Australian study of renal cancer predisposition syndromes to date, involving a cohort of 57 individuals affected by BHD (**Figure 1**) or HLRCC (**Figure 2**). Three patients had suspicious lesions identified during screening, all of whom had HLRCC. In two of these patients, the identified lesion was found to be RCC. We demonstrate feasibility of specialised and coordinated care for those affected by these rare disorders at scale and over time.

A new model of care was adopted to ensure implementation of national surveillance guidelines. Those with suspected genetic predisposition to RCC and their relatives were initially referred for clinical assessment by a cancer geneticist, with at-risk family members being subsequently offered predictive testing when appropriate. Affected patients were then cared for longitudinally by a nephrologist with a special interest in kidney genetics. This model of care also involved regular multidisciplinary team meetings and case conferences involving a cancer geneticist, nephrologist, urologist, and radiologist. Surveillance imaging was funded by the state public health service (Queensland Health) which likely improved compliance keeping in mind that in other Australian states and jurisdictions the cost of MRI may not be covered by the state health service with patients required to contribute to or self-fund such imaging costs. Elements of this model of care were adapted from a parallel genetic kidney disease clinical program (17). This tailored model ensured interconnected subspecialty expertise within a multidisciplinary context in regard to initial diagnosis and genetic counselling, longitudinal care and screening, and intervention if/when required.

The phenotypic features of our BHD subcohort were similar to those previously reported. Fibrofolliculoma is the most common cutaneous feature of BHD which usually develops after puberty. It was detected in 16/28 individuals, trichodiscoma in one of 28, and a further five of 28 had cutaneous changes suspicious for fibrofolliculoma. This prevalence of BHD cutaneous features was consistent with previously published data of up to 90% (18), keeping in mind that not all patients had undergone comprehensive assessment by dermatologist. Furthermore, pulmonary cysts are the second commonest feature of BHD and have been reported in up to 84% of patients, with spontaneous pneumothoraces in 22%–38% of patients (7, 18). In our cohort, pulmonary cysts were noted in 39% of patients and spontaneous pneumothorax in 28% of patients. Whilst the prevalence of pulmonary cysts was lower than previously reported, this is strongly suspected to be an underappreciation of asymptomatic pulmonary involvement and is likely related to the unavailability of baseline chest imaging in 10/28 individuals and to the low sensitivity of chest radiograph in detecting basal pulmonary cysts (19), as was undertaken in most BHD patients who had reviewable chest imaging. Most individuals with BHD who had pulmonary cysts (six of seven) and spontaneous pneumothorax (four of five) had disease-causing *FLCN* variants in exons 11 and 12, consistent with previous correlations between pulmonary features and pathogenic variants in exon 9 and greater (18, 20). There was no clear correlation between phenotype and ethnicity in this cohort.

Prevalence of RCC amongst those with BHD was 7% which is lower than in other studies (12%–34%) (4, 20, 21). This is likely to be related to the small size of our cohort and relatively short period of follow-up. By comparison, parotid oncocytoma is a rare feature of BHD and has been reported in at least nine cases in other cohorts (22–25). Here, we report two individuals with BHD who were diagnosed with parotid oncocytoma, neither of whom experienced complication from the oncocytoma itself or its management.

Again, whilst small, the genetic landscape identified in our BHD cohort adds to our understanding of this rare disorder. There were seven pathogenic (two of seven) or likely pathogenic (five of seven) heterozygous *FLCN* variants identified on clinical diagnostic genetic testing (**Table 3**) representing 23 of 28 patients. Amongst those pathogenic/likely pathogenic variants in *FLCN:* there was one canonical splice site variant (c.249+1_2delGCinsA), one exonic in frame shift deletion (c.469_471delTTC), one missense variant (c.763C>T), an intronic 3 bp deletion (c.1177-5_1177-3-del) expected to disrupt conserved splicing site, and three nonsense variants (c.1157C>G, c.1285dupC and c.1318_1334dup).Two further heterozygous *FLCN* missense VUSs were identified. This genetic diagnosis rate of 82% compares favourability to those reported previously with genetic diagnosis rates approaching 88% (18).

The clinical characteristics of our HLRCC subcohort were also similar to previous reports. Cutaneous leiomyoma was present in 65% and uterine leiomyoma in 44% which is higher than previously reported case series (26, 27). Cutaneous leiomyosarcomas are rarely, reported in patients with HLRCC (28–30). It is difficult to ascertain if the cutaneous leiomyosarcomas occurred due to transformation from leiomyoma. Here, we include one patient who was diagnosed with cutaneous leiomyosarcoma at a young age. The same patient was also diagnosed with RCC at 11 years of age and experienced uterine leiomyomas. Of note, her sister was also diagnosed with RCC at 19 years and her mother with RCC at 43 years. Overall, RCC was diagnosed in nine of 29 patients (31%) which was higher than previously reported lifetime risk of 15% (27). These findings in our cohort may have been due to ascertainment bias.

The genetic findings in this HLRCC cohort also add to those previously identified and are likely to be of assistance in variant assessment and clinical prognostication for further cases in the future. There were 12 pathogenic (10/12) or likely pathogenic (two of 12) *FH* variants identified. Both likely pathogenic variants were initially reported as VUS however were reappraised and reclassified based upon cosegregation evidence. A frameshift deletion in exon 4 of *FH* gene (c.413-414del, p.Lue138fs) was identified in three family members (H7, H8, H9) and was associated with early onset RCC (11, 19 and 43 years), cutaneous leiomyoma, and uterine leiomyoma in all affected family members, with one additionally experiencing cutaneous leiomyosarcoma. This variant was previously reported in a 44-year-old male who had RCC and had a twin brother who died from metastatic “kidney cancer” (31). No instances of smooth muscle tumour of uncertain malignant potential (STUMP) were identified or reported amongst this cohort.

There are several weaknesses with our study. Firstly, whilst a significant number of patients were identified there is a diagnosis bias towards those who either themselves experience symptoms or who have a relative who does. This is important given the variable lifetime penetrance of these rare disorders, unclear allelism within affected families, and the potential for one, several, or no associated phenotypes to occur within affected individuals. Secondly, whilst exhaustive efforts were undertaken to identify all diagnosis cases, there is a potential that some may not have been identified within our service. Furthermore, there is the significant potential for additional affected though undiagnosed patients to be present within our community. This may be due to the relative rarity of these disorders and paucity of awareness in regard to their features and potential diagnosis. The follow-up time of our cohort is also modest, and chest imaging was not applied commonly amongst those with BHD. This has meant that whilst our observed prevalence of symptomatic pulmonary involvement in concordant with previous reports, the prevalence of asymptomatic pulmonary involvement is much lower than indicated in other cohorts and is very likely to be a significant underrepresentation that is further compounded by the infrequent use of chest CT imaging. Lastly, even though our accessible clinical record systems provide comprehensive information of clinical encounters and investigations, there is potential that additional clinical encounters and/or screening investigations may not have been able to be identified.

Our study demonstrates the feasibility and early success of such an integrated approach to effective diagnosis, screening, and management for those affected by BHD and HLRCC. This model is now iteratively growing to provide service within the local public and universal healthcare system for the entirety of our state jurisdiction of approximately five million residents. Ongoing audit and quality assurance assessment of this model is indicated at regular intervals. Future consumer engagement and investigation of patient perspectives and experiences of this clinical service model will be key to its further evolution.

In addition to access to evidence-based screening service, one of the additional benefits is access to expert contribution to assessing findings on surveillance imaging and implementing evidence-based management strategies. Although established surveillance guidelines for RCC exist for those two conditions, those guidelines are for individuals who never developed RCC, and the assessment of many renal lesions detected on MRI can be challenging and require cautious assessment by an experienced radiologist. The presence of an experienced radiologist with special expertise in assessing renal lesions on MRI and an experienced urologist with a special interest in nephron-sparing surgeries is of great value to this multidisciplinary team. Several studies have demonstrated substantial improvement in overall quality of care and outcomes of several types of cancer in multidisciplinary team approach (32). Similar model of MDT approach in a cohort of patients with urological cancers has shown dramatic clinical impact on diagnostic and management decisions (33). Broader application of this model with a network of appropriate expertise is likely to be beneficial to other disease groups.

In summary, this study is the first and largest Australian clinical and molecular study of two rare renal cancer predisposition syndromes (BHD and HLRCC). We identified affected individuals (proband and relatives) based on molecular diagnosis. Although clinical diagnostic criteria for HLRCC and BHD exist (2, 4, 13), it was difficult to practically apply fibrofolliculoma (BHD) and cutaneous leiomyoma (HLRCC) as major diagnostic criteria as this was not confirmed on histology in the majority of the patients. In addition, RCC was the leading feature to raise the suspicion for BHD or HLRCC and molecular testing establishment of a clinical diagnosis in company with dedicated and multidisciplinary clinical assessment. This new model of care that has been adopted by our tertiary centre ensured effective diagnosis, longitudinal screening and follow-up, and prompt intervention when indicated for those affected with these rare renal cancer predisposing disorders. The success of this model is highly dependent on the experience of the team including a uroradiologist, experienced tertiary referral renal surgeon, clinical geneticist, and a nephrologist. Broader application of this model is likely to benefit patients where a network of such required expertise is available and a longitudinal multidisciplinary team is formed, even if virtually. Whilst the roles of clinical geneticists, genetic counsellors, radiologists, and urologists were separately well established, we demonstrate practical and patient-centric implementation of a new and emerging role (34, 35) for nephrologists in the management of renal tumours within a multidisciplinary setting.

## Author’s Note

AM received a RACP Jacquot Research Establishment Fellowship and MNHHS Clinical Research Fellowship.

## Data Availability Statement

The original contributions presented in the study are included in the article, further inquiries can be directed to the corresponding author/s.

## Ethics Statement

This study was approved by the Human Research Ethics Committee of the Royal Brisbane and Women’s Hospital (HREC/17/QRBW/276). As an ethically approved audit, informed consent was not required with all individual-level data being deidentified upon analysis and inclusion in all products of this study.

## Author Contributions

MA-S and AM conceptualised the study and undertook ethical approvals. MA-S and JW undertook data extraction with MA-S and AM undertaking analysis. HM, RS, SG, and SW contributed to study design and interpretation. MA-S and AM drafted the manuscript with all coauthors providing input, review, and edits. All authors contributed to the article and approved the submitted version.

## Conflict of Interest

AM has received research funding from Sanofi Genzyme and Otsuka, both outside this work.

The remaining author declares that the research was conducted in the absence of any commercial or financial relationships that could be construed as a potential conflict of interest.

## Publisher’s Note

All claims expressed in this article are solely those of the authors and do not necessarily represent those of their affiliated organizations, or those of the publisher, the editors and the reviewers. Any product that may be evaluated in this article, or claim that may be made by its manufacturer, is not guaranteed or endorsed by the publisher.
